# The potential for using aerated steam to eradicate strawberry mite and two-spotted spider mite on strawberry transplants

**DOI:** 10.1007/s10493-022-00757-0

**Published:** 2022-11-03

**Authors:** Nina Svae Johansen, Nina Trandem, Vinh Hong Le, Arne Stensvand

**Affiliations:** 1 Division of Biotechnology and Plant Health, Norwegian Institute of Bioeconomy Research, PO Box 115, 1431 Ås, Norway; 2 Faculty of Biosciences, Norwegian University of Life Sciences, PO Box 5003, 1432 Ås, Norway

**Keywords:** Cyclamen mite, *Phytonemus pallidus*, Phytosanitation, *Tetranychus urticae*, Thermotolerance, Vapour heat

## Abstract

In this study, we investigated if a steam treatment program used to produce disease-free strawberry transplants has the potential to also eliminate strawberry mite (*Phytonemus pallidus*) and two-spotted spider mite (*Tetranychus urticae*). Crowns of strawberry plants collected in a commercial field, containing young, folded leaves with all life stages of *P. pallidus*, and strawberry leaf discs on water agar with *T. urticae* with non-diapausing adult females and eggs from a laboratory rearing, were exposed to warm aerated steam in a steam cabinet in a series of four experimental runs over 2 years. The steam treatments constituted of a 1-h pre-treatment with 37 °C steam followed by a 1-h recovery period at 21–25 °C, and then a main steam treatment at 44 °C for either 2, 4 (both *P. pallidus* and *T. urticae*) or 6 h (the more heat tolerant *T. urticae* only). After steaming, the plant material with *P. pallidus* or *T. urticae* were incubated at 21–25 °C until survival was assessed after 1–6 days, depending on the mite species and life-stage. Non-steamed plant material with mites was used as controls. The 4-h treatment killed all *P. pallidus* eggs, larvae and adults, and the 2-h treatment killed all individuals in all three stages except for one egg in one of the runs. There were no or minor effects of the steam treatments on *T. urticae* adult and egg survival. Based on these results, the tested steam treatments may be used to eliminate the strawberry mite but not the two-spotted spider mite from strawberry planting material.

## Introduction

The strawberry mite (also named cyclamen mite or strawberry tarsonemid mite), *Phytonemus pallidus* Banks (Acari: Tarsonemidae), and the two-spotted spider mite, *Tetranychus urticae* Koch (Acari: Tetranychidae), are both serious cosmopolitan pests in strawberry (*Fragaria* × *ananassa* Duch) (Lindquist 1986; Cross et al. 2001; Migeon and Dorkeld 2021; Alford 2014). The two mite species prefer different parts of the strawberry plant.

*Phytonemus pallidus* is found in largest numbers on emerging, still folded leaves (Fitzgerald et al. 2008), reflecting its requirement for high humidity, especially for the juveniles (Wiesmann 1941). Its life cycle consists of egg, larva, pharate nymph (called pupa henceforth) and adult (Lindquist 1986). The development from oviposition to adult takes around 12 days at 20 °C (Easterbrook et al. 2003). At the end of the growing season, the adult females move into the crowns of the strawberry plants where they hibernate (Wiesmann 1941; Easterbrook et al. 2003). The population typically peaks in late summer. Heavily infested plants become stunted, reducing the yield (Alford 1972; Stenseth and Nordby 1976; Hellqvist 2002). The well-hidden lifestyle and small body size (≤﻿ 0.25 mm) of this species impede early detection as well as effective chemical control.

*Tetranychus urticae* prefers to oviposit and feed on the underside of mature and old leaves (Fitzgerald et al. 2008; Sudo and Osakabe 2011). Diapausing adult females tend to move to dark and sheltered places near the plant (Popov and Veerman 1996; Fitzgerald et al. 2008). Due to their feeding damage and high reproductive capacity, regular control actions need to be taken, particularly under warm and dry conditions (Tuovinen and Lindqvist 2010). The increasing practice of growing strawberry under cover in fields, tunnels and greenhouses in Europe has increased the problem with this species (Easterbrook et al. 2001). The mites’ preference for the leaf underside (Sudo and Osakabe 2011), and their high ability to develop acaricide resistance (Van Leeuwen et al. 2010), make chemical control challenging.

Both *P. pallidus* and *T. urticae* can be controlled by biocontrol agents, particularly predatory mites (Tuovinen 2000; Cross et al. 2001; Easterbrook et al. 2001; Tuovinen and Lindqvist 2010; Fountain and Medd 2015), but this is often hampered by chemical compounds used against other pests (Johansen and Trandem 2015). Establishing strawberry crops with mite-free plant material is of vital importance to alleviate later mite problems and reduce the number of chemical treatments and the risk of acaricide resistance development. Particularly *P. pallidus* (Hellqvist 2002; Tuovinen and Lindqvist 2010), but also *T. urticae* (Renkema et al. 2020) can be spread with infested strawberry transplants. As neither chemical nor biological control can be expected to eliminate *P. pallidus* or *T. urticae* in the transplant production, there is a great need for alternative methods to rid strawberry transplants of these mites before planting (Tuovinen and Lindqvist 2010; Kruistum et al. 2012; Renkema et al. 2020).

It has been known for several decades that hot water treatment of strawberry plant material can eliminate *P. pallidus* (Hodson 1934; Smith and Goldsmith 1936; Stenseth 1975; Tuovinen 2000; Hellqvist 2002). According to Stenseth (1975) in Norway all adults and eggs on runners were killed within 8 and 4.5 min when immersed in 44 and 46 °C water, respectively. In Sweden, Hellqvist (2002) found that treatment in water at 44, 46 or 48 °C followed by 5 min at 30 °C eradicated adults on leaflets after 6.0, 2.5 and 1.3 min, respectively. In a study in Finland with adult females and males, larvae and eggs of *P. pallidus* on runners, eradication was achieved after 5–10 min in 46.5 °C water, whereas female survival was 1% at 45 °C and 10% at 43.5 °C (Tuovinen 2000).

Another way to heat plant material is to use steam. Preliminary experiments in Norway showed that treatment of potted strawberry runners with 30 or 60 min of aerated steam at 46 °C in a prototype steam cabinet killed almost all *P. pallidus* present in the plants (Stensvand et al. 1999). Tuovinen et al. (2003) from Finland reported that all strawberry mites on potted runner plants were killed if the air temperature and relative humidity (RH) were kept at 43 °C and 80–100%, respectively, for 42 min after a warming-up period of 30 min. Warm vapour treatment was also tested by Smith and Goldsmith (1936) in California, finding that 45 min at 43.3 °C killed all cyclamen (strawberry) mites in heavily infested strawberry plants, whilst 25 min left some mites alive.

Some studies have evaluated the potential for using heat to eradicate *T. urticae* on plant material. Hot water dipping of persimmon fruits at 44–54 °C (Lester et al. 1997) and strawberry leaf discs at 47.5–57 °C (Gotoh et al. 2013), warm air treatment of persimmon fruits at 43.2–50 °C (Cowley et al. 1992), nectarine fruits at 43.2–48.2 °C (Waddel and Birtles 1992), and aerated steam treatment of bean leaf discs at 44–48 °C (Renkema et al. 2020) can eradicate or reduce survival of eggs (Gotoh et al. 2013; Renkema et al. 2020), non-diapausing females (Cowley et al. 1992; Waddel and Birtles 1992; Lester et al. 1997; Gotoh et al. 2013; Renkema et al. 2020) and diapausing females (Waddel and Birtles 1992; Lester et al. 1997). Hence, heat treatment has been suggested as a possible method to manage *T. urticae* on plant material (Waddel and Birtles 1992; Lester et al. 1997), including strawberry seedlings, runners and transplants (Gotoh et al. 2013; Renkema et al. 2020).

In experiments with aerated steam treatments of strawberry transplants in Florida, USA, it was shown that 37 °C pre-treatment followed by 1 h at ambient temperature and no steam stimulated the formation of heat shock proteins in the transplants so that they would tolerate 4 h at 44 °C without yield reduction (Brown et al. 2016; Wang et al. 2019). This treatment has strongly reduced or eliminated pathogens such as the bacterium *Xanthomonas fragariae* and the fungi *Colletotrichum acutatum*, *Botrytis cinerea* and *Podosphaera aphanis* from strawberry transplants (Zuniga and Peres 2016, 2017; Wang et al. 2017; Da Silva et al. 2019; Turechek et al. 2021) and is currently recommended as a phytosanitary program to control plant pathogens in strawberry transplants (Turechek et al. 2021). In experiments including bare root plants in Florida (Wang et al. 2019; Turechek et al. 2021) and plug (tray) plants in Norway (Nielsen 2021), there were no negative effects on growth and yield reported by using this program to steam strawberry transplants. In this study, we investigated the potential to use the ‘Florida’ treatment program to control *P. pallidus* and *T. urticae* in planting material of strawberry.

## Material and method

**Fig. 1:**
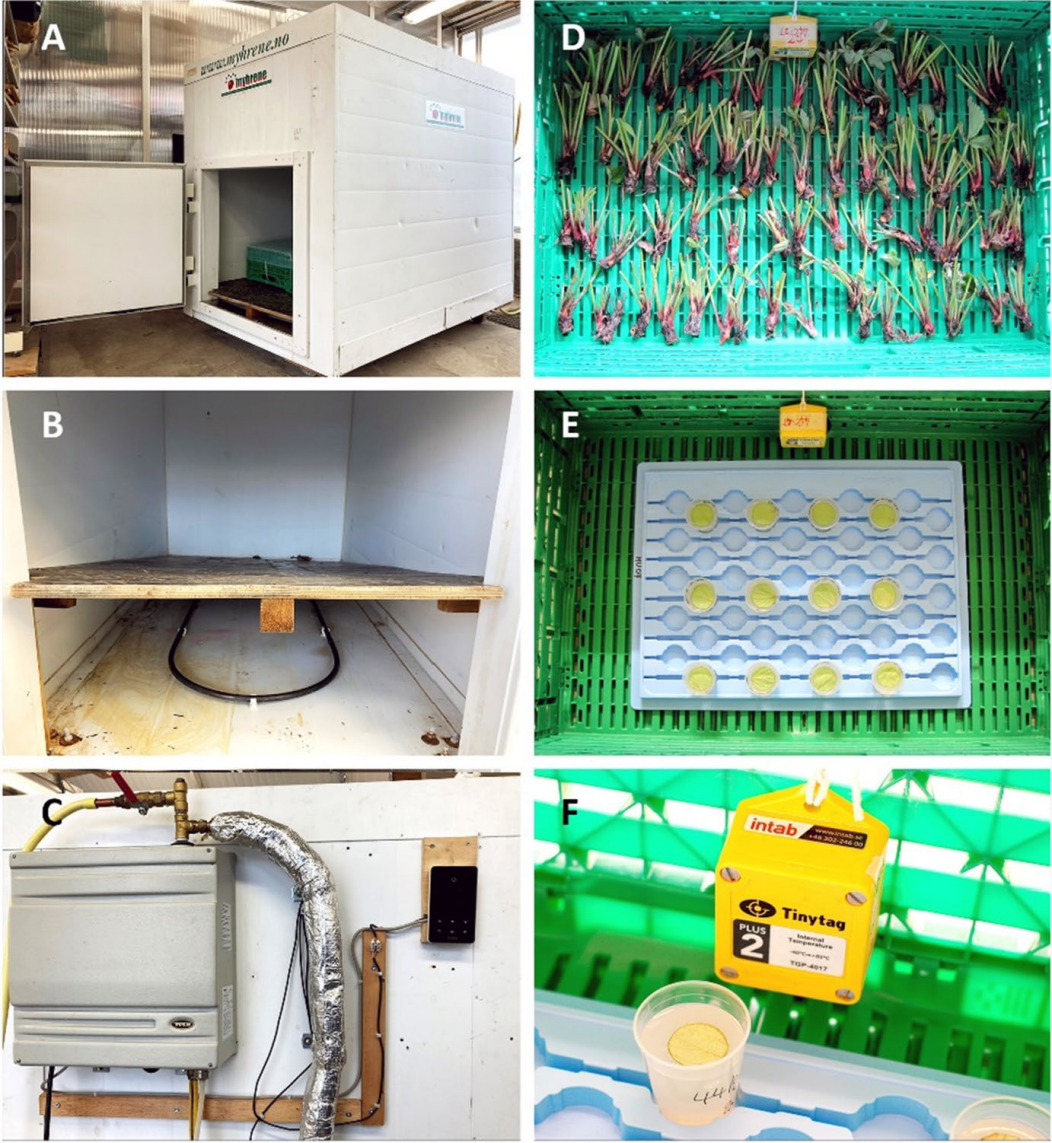
The steam cabinet and setup used for the steaming experiments. **A** The steam-cabinet was separated into two rooms with a wood fibreboard—the lower antechamber at the basement and the upper steam treatment chamber. **B** The lower antechamber containing the perforated steam pipe that leads the steam into the cabinet. **C** The steam generator (grey box on the left), the temperature regulator (dark screen on the right) and the insulated steam pipe connected to the lower antechamber. **D** Strawberry crowns with 1–2 young, folded trifoliate leaves carrying different life stages of *Phytonemus pallidus*. **E** Strawberry leaf discs with *Tetranychus urticae* female adults and eggs placed on water agar in plastic cups. **F** One of the Tinytag temperature loggers

The effect of exposure to 44 °C aerated steam on survival of *P. pallidus* and *T. urticae* on strawberry plant material was studied in a steam cabinet (Moleda AS, Sylling, Norway; Fig. 1A) placed in a greenhouse compartment at the Centre for Plant Research in Controlled Climate, in Ås municipality (South-East Norway). There was a series of four runs, two in August 2019 (run 1 and 2) and two in August 2020 (run 3 and 4). Both mite species were included in all runs (Table 1).

**Table 1:** Overview of the mite species and plant material used in the various steam treatments and in the non-steamed control in the four runs in the steam cabinet. Plant material with both mite species was included in all runs

Run no. (year)	Mite species	Amount and type of plant material with mites			
		Main steam treatment at 44 °C^a^			Untreated control (0 h)
		2 h	4 h	6 h	
1 (2019)	*Phytonemus pallidus*	10 Crowns + 10 single leaves	10 Crowns + 10 single leaves	–	10 Crowns + 10 single leaves
	*Tetranychus urticae*	12 Leaf discs	12 Leaf discs	–	12 Leaf discs
2 (2019)	*P. pallidus*	10 Crowns	10 Crowns	–	10 Crowns
	*T. urticae*	12 Leaf discs	12 Leaf discs	–	12 Leaf discs
3 (2020)	*P. pallidus*	18 Crowns	18 Crowns	–	18 Crowns
	*T. urticae*	–	12 Leaf discs	12 Leaf discs	12 Leaf discs
4 (2020)	*P. pallidus*	36 Crowns	36 Crowns	–	36 Crowns
	*T. urticae*	–	12 Leaf discs	12 Leaf discs	12 Leaf discs

^a^The plant material had been pre-treated with 37 °C steam for 1 h and cooled down at 21–25 °C for 1 h before each of the main steam treatments

### Origin of mites and plant material

#### *Phytonemus pallidus*

In both of the 2 years, whole strawberry plants with *P. pallidus* symptoms were collected from a commercial perennial strawberry field crop, planted the previous year, in Eidsberg municipality (South-East Norway) at the end of harvest, just before acaricide application. Twelve plants of cv. Faith were collected on 13 August 2019, 35 plants of cv. Flair were collected 11 August 2020 and 12 plants of cv. Malwina were collected 18 August 2020. Each plant had multiple crowns. In 2019, approximately 100 single young, folded trifoliate leaves were collected as well. The plant material was transported to Norwegian Institute of Bioeconomy Research (NIBIO) in Ås municipality (South-East Norway), where the presence of *P. pallidus* on the whole plants and single leaves was verified in the laboratory. To avoid disturbing the mites and their preferred natural habitat of young, folded leaflets, they were not counted before steaming because this could have affected the outcome of the treatment in the steam cabinet. Until the steam treatments took place, all plant material was kept outdoors, out of the sun at natural day-length and temperature range for the season (12–21 °C recorded at a weather station 300 m away). The single young, folded leaves were kept in small zip bags (10 × 15 cm) for 1 day before they were used in run 1. The whole plants were kept singly or pairwise in transparent, ventilated plastic bags (50 × 88 cm) for 1 day (run 1) or 7 days (remaining runs) before steaming. All plant material was kept moist by adding water as needed to avoid wilting of the plants and to keep the high humidity required for *P. pallidus* survival.

#### *Tetranychus urticae*

*Tetranychus urticae* was reared on strawberry plants of cv. Korona in an indoor insectary without windows at 22 ± 0.5 °C and 60–70% RH at NIBIO. A daily photoperiod of 16 h light (75–95 μmol m^−^^2^ s^−1^ PAR) was provided with fluorescent tubes (TL-D 90 Graphica 36 W 965, Philips, The Netherlands). This strain was originally collected on *Aspidistra* sp. in South-East Norway in 2000, reared on bean (*Phaseolus vulgaris*) until 2015 and since then on strawberry. Strawberry plants of cv. Korona were grown in a growth chamber at NIBIO with the same climate and light conditions as in the insectary.

### Preparation of plant material with mites for steaming

Plant material with *P. pallidus* or *T. urticae* to be treated in the steam cabinet was prepared in the morning at the same day as the steaming took place.

#### *Phytonemus pallidus*

For each of the four runs in the steam cabinet, whole strawberry plants with *P. pallidus* were retrieved from the plastic bags stored outdoor. Each plant was split into 10–20 crowns (according to number of crowns on the plants) and stripped of roots and unfolded leaves, leaving mainly 1–2 young, folded trifoliate leaves (i.e., 3–6 single leaflets) attached to them. Out of these, 30, 30, 54 and 108 crowns were randomly selected for use in the four runs, respectively (Table 1). For each run, the selected crowns were randomly distributed in equal numbers at the bottom of three perforated Green Plus 6413 trays (height 13 cm, width 40 cm, depth 60 cm; IFCO Systems, Pullach, Germany): one tray for each of two steam treatment times (2 and 4 h) and one for an untreated control (0 h) (Table 1). The crowns were placed so they did not overlap to ensure that all plant parts were exposed to the steam (Fig. 1D).

In addition, for run 1, 30 single young, folded leaves with *P. pallidus* were randomly selected from the zip-bags stored outdoor. Each of the leaves was placed in a Petri dish (diameter 14 cm) without lid and distributed in three Green Plus trays for the same treatments as the crowns (0, 2 and 4 h).

#### *Tetranychus urticae*

For each of the four runs, 36 leaf discs with diameter 2 cm (3.14 cm^2^) were cut from unfolded, mature leaves (the preferred habitat of *T. urticae*) of strawberry plants of cv. Korona grown in the growth chamber as mentioned above. The leaf discs were placed singly with the abaxial side facing upward on 1.5% water agar in 30-mL plastic cups, as *T. urticae* prefer this side of the leaves. Strawberry leaves with *T. urticae* were then obtained from the mite rearing in the insectary. Mite cohorts were established on the leaf discs by carefully transferring 10 (run 1 and 2) or 5 (run 3 and 4) randomly selected adult females (mating status unknown) from the leaves to each of the leaf discs with a fine brush and leave them to oviposit for 5 days in the insectary.

The 5th day, 1–3 h before the steaming took place, the eggs produced by the females were counted. The mean (± SD) number of eggs per leaf disc was at that time 128.7 ± 35.1 in run 1, 105.4 ± 23.4 in run 2, 121.4 ± 16.0 in run 3 and 92.8 ± 18.7 in run 4. Adult female survival was checked as well, and dead females were removed from the leaf discs and later excluded from the statistical analysis. Thus, the corrected mean (± SD) number of adult females per leaf disc was 9.7 ± 0.5 in run 1, 9.2 ± 0.9 in run 2 and 5.0 ± 0.0 in runs 3 and 4. After counting, 12 plastic cups with adults and eggs were randomly distributed to each of three Green Plus trays, one tray for the untreated control (0 h) and one tray for each of two steam treatment times (Table 1). In run 1 and 2 in 2019, treatment times of 2 and 4 h were selected, but after evaluation of the mortality of the steamed mites we decided to increase the treatment times to 4 and 6 h in run 3 and 4 in 2020. The plastic cups were without lids during steaming to ensure good exposure of the mites to the steam.

### Steam cabinet construction and general principle of temperature regulation

The steam cabinet used in the runs (Fig. 1A) had a total inside volume of 1.66 m^3^ (height 118 cm, width 94 and depth 150 cm). It constituted two chambers that were horizontally separated by a 1.5-cm-thick wood fibreboard: a lower antechamber of 0.07 m^3^ (height of walls 4.7 cm, floor 94 × 150 cm) at the base where the steam entered the cabinet, and an upper steam treatment chamber of 1.58 m^3^ (height of walls 112 cm, floor 94 × 150 cm) where the plant material was placed during steaming. The lower antechamber had a 15-mm-diameter copper pipe (Fig. 1B) attached to a 12 kW steam generator (10 kOhm; Tylö, Halmstad, Sweden) (Fig. 1C). The copper pipe had 10 circular openings (2.5–6.0 mm diameter) where the steam left the pipe and distributed in the antechamber and raised further up into the steam treatment chamber through 2-cm-wide slits along each of the long sides of the wooden fibreboard. The diameter of the openings in the pipe increased with the distance from the generator to compensate for the loss of steam pressure, providing an even distribution of the steam in the chamber during steaming. The steam chamber had 12.5-cm-thick insulated walls to minimize heat loss during steaming. There was no light source, thus, when the plant material was placed in the chamber and the door was shut, the steam treatment took place in complete darkness.

The general principle of temperature regulation in the steam cabinet was as follows: The plant material to be steamed was placed in the upper steam treatment chamber. A set temperature was selected, and the steam generator started. The temperature of the steam leaving the generator was 100 °C, and when released into the cooler air in the lower antechamber, the air became water saturated and warmed up due to heat transfer from the steam. As the air temperature increased, the water-saturated air raised through the slits in the wooden fibreboard and into the steam treatment chamber where it again met cooler air, condensed, and transferred heat to the air and to the surfaces of the plant material and mites which remained wet due to condensation throughout the steam treatment period. An electronic sensor recorded the temperature in the lower antechamber and regulated the steam production in the generator. The steam generator temporarily stopped steam production when the selected set-temperature was reached and started again when temperature in the chamber decreased due to loss of heat to the environment—in this way the temperature in the steam treatment chamber was balanced within ± 0.5 °C.

### Steam treatments used for *Phytonemus pallidus* and *Tetranychus urticae*

The same steaming program was used in all the four runs and consisted of four successive steps. Step 1, introduction of plant material: two trays with strawberry crowns with *P. pallidus* (and two trays with single young folded, trifoliate leaves included in run 1) and two trays with plastic cups with leaf discs on water agar containing *T. urticae* females and eggs (prepared for steaming as described above) were placed on the floor in the upper steam treatment chamber. Step 2, pre-treatment: the temperature was set to 37 °C, the generator started to produce steam and the temperature increased until the set temperature was reached and then balanced within ± 0.5 °C for 1 h. Step 3, cooling down: the steam generator was stopped and all the trays were removed from the steam treatment chamber and placed outside the cabinet were they were kept for 1 h at the ambient room temperature (21–23 °C in run 1, 22–24 °C in run 2, 23–25 °C in run 3 and 21–23 °C in run 4). The aim of this pre-heating and cooling was to increase the heat tolerance of plants. Step 4, main treatment: the trays were reintroduced to the steam treatment chamber, the temperature was set to 44 °C, the generator started to produce steam and the temperature increased until the set temperature was reached and then balanced within ± 0.5 °C for the rest of the steam treatment period. Steaming periods of 2 and 4 h were used for both mite species in the two runs in 2019: when the plant material had been steamed at 44 °C for 2 h, the steam cabinet door was opened, half of the steamed plant material with *P. pallidus* (one tray) and half of that with *T. urticae* (one tray) were quickly removed from the chamber and the door was shut again, leaving the remaining plant material (one tray with *P. pallidus* and one tray with *T. urtice*) to be steamed at 44 °C for another 2 h (i.e., 4 h steaming in total). The door was kept open for < 30 s.

After evaluation of the mortality of the steamed mites in the two runs in 2019, the same treatment times (2 and 4 h) were selected for *P. pallidus* in runs 3 and 4. *Tetranychus urticae* seemed, however, to be more heat resistant, so the treatment times for this species were set to 4 and 6 h in runs 3 and 4. In practice, this meant that one tray of the plant material with *P. pallidus* was removed from the steam treatment chamber after 2 h and one tray after 4 h, whereas the first tray of the plant material with *T. urticae* was removed after 4 h and the second tray after 6 h.

In each run, the trays with plant material with mites to be exposed to the different treatments were randomly placed on the floor of the steam treatment chamber to avoid any bias related to steam distribution in the chamber or distance to the steam cabinet door. The non-steamed controls, one tray for each of the mite species, was placed on the floor outside the steam cabinet during each run.

One Tinytag Plus 2 (TGP-4017; Gemini Data Loggers UK, Chichester, UK) was mounted close to the treated plant material in each tray, which recorded the air temperature every minute (2019) or every third minute (2020) throughout all four runs as shown in Fig. 1D–F.

### Determining survival of *Phytonemus pallidus* and *Tetranychus urticae* after steaming

#### *Phytonemus pallidus* adults and larvae

Survival of *P. pallidus* larvae and adults was studied in runs 1, 3 and 4 (in run 2 only a pilot study of egg survival was done as described below). Within 30 min after each run was finished, the steamed and non-steamed crowns with 1–2 young, trifoliate, folded leaves (all runs) and the single young, folded trifoliate leaves (run 1 only) were put in sealed plastic bags (30 × 50 cm, all plant material from each treatment in one bag) and kept at 21–25 °C. Two days (runs 1 and 4) or 3 days (run 3) after steaming, the live and dead adults and larvae were counted under a stereomicroscope (115 × magnification) on 28–30 randomly selected leaflets picked from the young folded trifoliate leaves on the crowns (all runs) and the single leaves (run 1 only). Individuals were categorized as dead if they did not move when carefully poked with a pin. In run 1, eggs and pupae were counted under the stereomicroscope as well, but without assessing survival, as these stages are immobile and it is impossible to determine whether they are dead or not with certainty.

#### *Phytonemus pallidus* eggs

A pilot study of egg survival when transferring eggs from mite-crowded leaves to mite-free leaf discs was performed in run 2, as following the fate of single eggs is difficult on leaflets with many mites. Strawberry crowns with young, folded leaves was treated in the steam cabinet, and leaflets were randomly selected from the crowns as described above for the other runs. In total, seven and 11 eggs steamed for 2 and 4 h, respectively, and 28 non-steamed eggs (0 h) were included. Six leaf discs (diameter 2 cm, 3.14 cm^2^) were cut from mature folded leaves of strawberry plants of cv. Korona grown in the growth chamber described above. These were placed with the abaxial side upwards on 1.5% water agar in 30-mL plastic cups, and the eggs found on the steamed and non-steamed leaflets were carefully transferred to the leaf discs (eggs from one treatment on 1–3 leaf discs) with a one-haired brush. The plastic boxes were sealed with ventilated lids to keep a high humidity and incubated in a climate chamber (MLR-352H-PE; PHC, Ora-gun, Japan) at 22 ± 0.5 °C, 60% RH, and a 16 h photoperiod provided with fluorescent tubes (Panasonic FL40SSENW37, Japan). The number of larvae that emerged from the eggs was checked under the stereomicroscope 8 days after steaming.

This method, slightly modified, was used in runs 3 and 4: leaflets not used for counts of adults and larvae were searched for eggs. Eggs were carefully transferred to the abaxial side of strawberry leaf discs (diameter 2 cm, 3.14 cm^2^; 21 leaf discs per run, 10 eggs per leaf disc) placed singly on water agar in plastic cups. To simulate the conditions where eggs normally are found on the plants, another leaf disc of similar size was gently placed with the abaxial side facing downwards on top of the eggs. Eggs from the mid and side leaflets of trifoliate leaves were kept on separate leaf discs in case those on mid leaflets had been better protected from the heat. Six to nine such leaf disc pairs were prepared per treatment and run. Because the pilot study showed that there was too much condensation of water on the leaf discs when they were incubated in the climate chamber, we incubated the eggs in runs 3 and 4 in the laboratory, at 21–25 °C and 30–77% RH. The temperature and humidity were recorded throughout the incubation period by a Tinytag Plus 2 logger (TGP-4500, Gemini Data Loggers, UK). Number of hatched eggs was recorded for each leaflet in the stereomicroscope 6 days after steaming, as the egg developmental time on strawberry has been found to be 4.7 days at 20 °C (Easterbrook et al. 2003).

#### *Tetranychus urticae*

Within 30 min after each run was finished, the steamed and non-steamed trays containing plastic cups with leaf discs with *T. urticae* female adults and eggs were placed in the climate chamber (MLR-352H-PE) at 22 ± 0.5 °C, 60% RH and L16:D8 photoperiod (50 µmol m^−2^ s^−1^ illumination provided by fluorescent tubes, Panasonic FL40SSENW37). One day after steaming, the live and dead adults, unhatched eggs, and live and dead larvae (more mature stages were not developed yet) per leaf disc were counted under a stereomicroscope (×115 magnification) in all four runs. After counting, each leaf disc with mites was transferred to a new 30-mL plastic cup with a fresh leaf disc (without mites) cut from the strawberry plants in the growth chamber to provide food of good quality for the mites. The steamed leaf disc slightly overlapped the fresh one to allow the mites to walk to the fresh leaf discs by themselves. The plastic cups with mites on leaf discs were then reintroduced into the climate chamber for further incubating. A pipette was used to place a thin layer of water on the water agar surface around, but not onto, the leaf disc every day during incubation to keep the leaf disc fresh and prevent the mites from escaping. Five days (runs 1 and 2) or 6 days (runs 3 and 4) after steaming, the live and dead larvae, nymphs (proto- and deutonymphs pooled) and adult females were counted. Individuals that did not move or clearly showed abnormal walking behaviour after being carefully poked with a needle were recorded as dead. Unhatched eggs and individuals in the chrysalis stages (proto-, deuto- and teleiochrysalis pooled) were counted as well, but without assessing survival as these stages are immobile and it is impossible to determine whether they are dead or not with certainty.

### Statistical analysis

#### *Phytonemus pallidus*

The data for *P. pallidus* were analysed separately for each of the four runs. Survival of the mobile stages (adults and larvae) was calculated as the mean proportion live individuals out of the total number of individuals in that stage per leaflet. Leaflets without any individuals of that particular stage were excluded from the analysis. Egg survival was calculated as the proportion of eggs that hatched out of the total number of 10 eggs per leaflet. The survival was used to estimate standard error (SE) for proportions. As the steaming killed all individuals but one single egg, the results for *P. pallidu*s were not further analysed statistically.

#### *Tetranychus urticae*

The data from 2019 and 2020 were analyzed separately as two different experiments (hereafter named 2019 and 2020), each year with the two runs as replicates in time. The probability of female adults to be alive on days 1 and 5 (2019) and days 1 and 6 (2020) after the steam treatments was analyzed with a logistic probability model for binomially distributed response variables, with steam treatment time as fixed factor, run as random factor, and with 12 observations of number of live and dead adults (12 leaf discs) within each treatment and run. Type III tests of fixed effects were used to test for significance of differences between treatments. If significant, Tukey–Kramer's method was used to group the treatments based on least square means. Significance level 0.05 was used in all tests and proc glimmix in SAS v.9.4 was used for calculations (SAS Institute, Cary, NC. USA).

The number of *T. urticae* eggs that had successfully hatched and produced a larva was estimated from the number of live and dead individuals in the larval or older life stages that in sum was present on each leaf disc on days 1 and 5 (2019) or on days 1 and 6 (2020). Egg survival was then calculated as the percentage of hatched eggs out of the initial number of eggs present per leaf disc before steaming. At each day of counting, survival in the mobile immature stages (larvae, protonymphs and deutonymphs pooled) was calculated as the percentage of live individuals out of the total number of individuals in these life stages per leaf disc. Furthermore, at the days of counting the percentage of individuals in the chrysalis stages (proto-, deuto- and teleiochrysalis pooled) out of the total number of live and dead individuals in all the developmental stages from larvae to teleiochrysalis per leaf disc was calculated.

As very few eggs had hatched the day after treatment in both years, and only newly emerged larvae were found, the data for egg hatching and larval survival at this time were not analyzed statistically. The response variables % egg survival, % survival in the mobile immature stages, % individuals in the chrysalis stages, and total number of immatures (eggs to teleiochrysalis) on day 5 (2019) or day 6 (2020) after steaming were analyzed using a mixed effects model with steam treatment time as fixed factor and run and the interaction between run and treatment as random factors, and with 12 observations (12 leaf discs) within each treatment and run. If the test of fixed effects showed significant differences between the treatments, Tukey’s pairwise comparisons with 95% confidence interval (CI) were used to test differences between each treatment. Number of live adults on days 1 and 5 (2019) or days 1 and 6 (2020) after steaming was included as covariates in the analysis of the number of eggs and total number of immatures (Minitab 19.2, 64-bit).

## Results

### Temperature during steaming

For both runs in 2019 it took ca. 25 min from trays with the plant material was placed in the steam treatment chamber for pre-treatment (step 2) before the air temperature reached 36 °C, after which the temperature stabilized between 36 and 37 ± 0.5 °C. When the steam generator temporarily stopped and the trays were removed from the steam chamber, the temperature decreased to ca. 24 °C. When the trays were reintroduced to the steam chamber and the main steam treatment started, it took ca. 25 min for the air temperature to reach 44 °C, whereafter the temperature stabilized around 44 ± 0.4 °C for the remaining treatment times.

For the pre-treatment in both runs in 2020, it took ca.  36 min to reach 37 °C, whereafter the temperature stabilized around 37 ± 0.5 °C. Following the period of 60 min when the trays with plant material were placed outside the steam cabinet  and the temperature decreased to around 27 °C, it took ca. 30 min to reach 44 °C, after which the temperature stabilized around 44 ± 0.4 °C for the remaining time of the experiment.

In all four runs, there was a minor and brief temperature drop when the steam chamber door was opened to remove the plant material that was treated for the shortest time.

### *Phytonemus pallidus*

#### Adult and larval survival

**Fig. 2:**
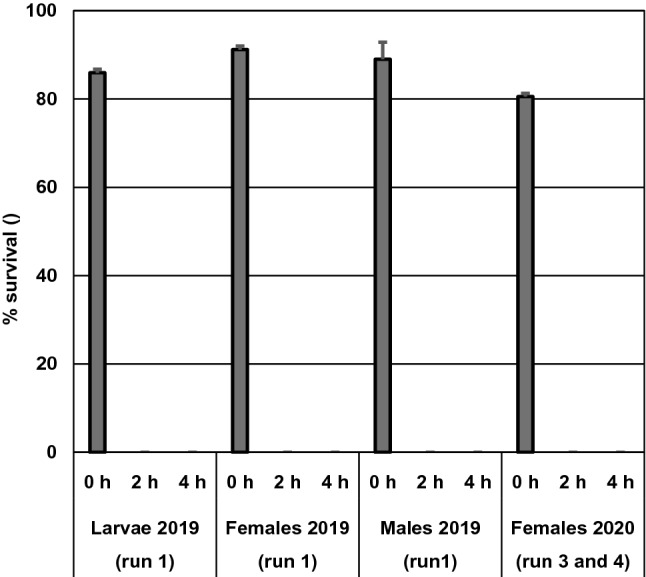
Mean (+ SE) survival (%) of *Phytonemus pallidus* larvae, adult females and adult males per leaflet assessed 2–3 days after treatment with 44 °C steam for 0, 2 or 4 h. 2019: survival only studied in run 1; means are based on 28 or 30 folded leaflets. 2020: values for two runs (3 and 4) are pooled, no males found, larval survival not fully recorded; means are based on 58 or 60 folded leaflets

In total for run 1, 3 and 4, 6201 individuals of *P. pallidus* in mobile stages (69% of them adult females, 1% adult males and 30% larvae) were counted and categorized as live or dead 2–3 days after steaming. No live larvae or adults were found on the leaflets steamed for 2 or 4 h whereas 81–91% were alive in the non-steamed control (Fig. 2). The mite survival on the leaflets attached to the crowns and on the leaflets picked from the single folded leaves in the Petri dishes were similar in run 1 (data not shown); thus, we only used crowns in the next three runs. Live and dead larvae were not counted in runs 3 and 4 in 2020 due to a very high mite density on the leaflets, but all steamed leaflets were carefully examined for live larvae, and none was found. The mean number of larvae, adult females and adult males per leaflet ranged from 11.1 to 28.1, 10.5 to 20.6 and 0.0 to 1.1, respectively. Except for larvae in the 2-h treatment in run 1, the total number of live and dead mobile individuals in the non-steamed controls was 1.4–2.2 × higher than in the steam treated leaflets.

#### Egg hatchability

**Fig. 3:**
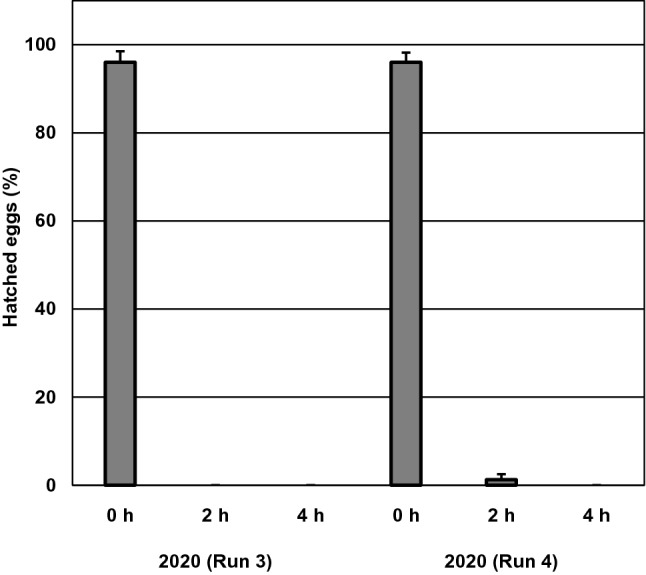
Mean (+ SE) hatchability (%) of *Phytonemus pallidus* eggs assessed 6 days after treatment with 44 °C steam for 0, 2 or 4 h in the two runs in 2020. Eggs from mid and side leaflets are pooled. Means are based on 6–9 egg batches per treatment (10 eggs per batch)

In the pilot study (run 2 in 2019), the observed egg hatching success was 54% in the untreated control (28 eggs) 8 days after steaming, whereas no eggs had hatched in the steam treatments (seven and 11 eggs from the 2- and 4-h treatments, respectively). In the two runs in 2020, in total 450 eggs were included. Except for five eggs in the untreated control, all eggs were accounted for, either as still unhatched eggs or larvae, 6 days after steaming. The unhatched eggs were considered dead. The five missing eggs were excluded from the calculation of survival. No eggs out of 170 survived in the 4-h treatment, whereas in the 2-h treatment, one single egg out of 140 hatched. This egg originated from a leaflet positioned in the middle of a folded trifoliate leaf. The hatchability in the control treatment was 96% in both runs (Fig. 3).

### *Tetranychus urticae*

#### Adult survival

**Fig. 4:**
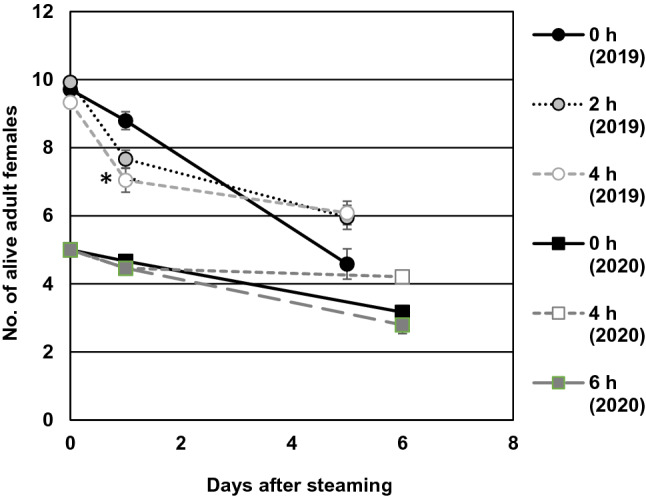
Mean (± SE, n = 12 leaf discs) number of live adult females of *Tetranychus urticae* assessed 1 and 5 (2019) or 1 and 6 (2020) days after steam treatment for 0, 2, 4 or 6 h at 44 °C. The asterisk (*) indicates statistical difference from the untreated control (0 h) (Tukey–Kramer's method, *p* < 0.05)

In 2019, steaming for 4 h significantly reduced the probability of an adult female being alive the day after treatment compared to the untreated control (*p* < 0.001, Fig. 4); however, no effect of steaming was found after 5 days (*p* = 0.13). In 2020, steaming for 4 or 6 h did not affect the probability of a female being alive 1 or 6 days after treatment (*p* = 0.56 and 0.25, respectively; Fig. 4).

#### Egg hatchability

**Fig. 5:**
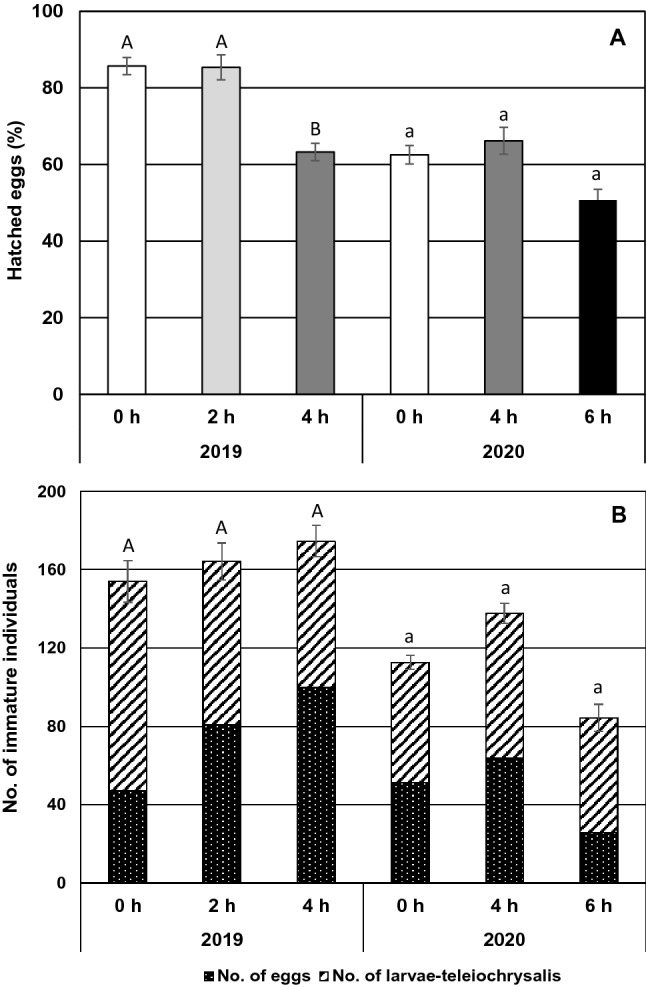
Mean (± SE, n = 12 leaf discs) (**A**) hatchability (%) of *Tetranychus urticae* eggs and (**B)** total number of immature (egg to teleiochrysalis) individuals assessed 1 and 5 (2019) or 1 and 6 (2020) days after treatment with 44 °C steam for 0, 2, 4 or 6 h. Means within an experiment capped with different letters are significantly different (Tukey’s pairwise comparisons: *p* < 0.05)

In 2019, steaming for 2 h did not affect the proportion of eggs that had hatched after 5 days, but 4 h steaming reduced the hatchability by 26% compared to the control (*p* = 0.037, Fig. 5a). Steaming for 4 or 6 h in 2020 did not significantly affect the proportion of eggs hatched after 6 days (*p* = 0.44), although a tendency for reduced egg hatching was seen in the 6-h treatment.

#### Larvae to teleiochrysalis survival

The first larvae were found 1 day after steaming in both years. The mean (± SE) number of larvae per leaf disc at that time was very low (2019: 0.2 ± 0.1; 2020: 12.4 ± 0.6), and no individuals had yet entered the protochrysalis stage. Steaming had no effect on the proportion of live larvae, protonymphs and deutonymphs (the three stages pooled before analysis) or on the proportion of individuals in the chrysalis stages (proto-, deuto- and teleiochrysalis pooled before analysis) assessed after 5 days in in 2019 (*p* = 0.58 and 0.51, respectively) or after 6 days in 2020 (*p* = 0.79 and 0.57, respectively). At the time of assessment, 98–100% of larvae, proto- and deutonymphs were still alive in all treatments in both experiments (data not shown). Whether the individuals in the chrysalis-stages were dead or alive at the time of counting was not possible to determine.

#### Development after steaming

Steaming of adult females and the 1–5 days old eggs had no impact on the total number of immature individuals (egg to teleiochrysalis stages) per leaf disc counted 5 (2019, *p* = 0.96) or 6 (2020, *p* = 0.33) days after treatment (Fig. 5b). The tendency for a slightly increased population density in the 2- and 4-h steam treatments in 2019 was mainly due to higher numbers of eggs in the populations and was explained by the covariate ‘number of mothers alive on day 5’ (*p* = 0.004). The covariate ‘number of mothers alive on day 6’ also accounted for the tendency for higher egg production (*p* = 0.014) and total number of immatures (*p* = 0.024) in the 4-h treatment in 2020. More than 93% of unhatched eggs, most of them presumably laid after treatment, had a normal appearance by visual inspection, but we could not record whether they were dead or not with certainty. No differences were found in number of individuals in the chrysalis stages (the three stages pooled) between the treatments. The mites had not reached adulthood of the next generation at the time of counting.

## Discussion

The steam treatment program developed in Florida (USA) to suppress plant pathogens on strawberry transplants that included a 1-h pre-treatment at 37 °C followed by 1 h at ambient air temperature, which in our runs was 21-25 °C, and then 4 h at 44 °C (Brown et al. 2016; Turechek et al. 2021), eradicated eggs, larvae and adults of the strawberry mite (*P. pallidus*) in the present experiments. Furthermore, if the 44 °C treatment ended after 2 h, no larvae and adults and only one egg in one of the runs survived. This shows the great potential in using steam treatments against this harmful strawberry pest. We did not count live and dead strawberry mites before steaming, as this would have required forcing the folded leaves open and thus damaging the mite habitat. We can, however, make the reasonable assumption that the average mite viability initially was the same across treatments, given the randomization of plants made before the steaming. For the two-spotted spider mite (*T. urticae*), there were no or only minor effects of the steam treatments on survival of eggs and adults.

When dipping strawberry runners and leaflets in warm water, *P. pallidus* was eradicated already after 6–12 min of exposure at 44 °C (Stenseth 1975; Hellqvist 2002). Although we did not test shorter exposure times than 2 h, parts of a discrepancy in time between hot water dipping and steaming necessary to kill the various stages of *P. pallidus*, may be explained by the ca. 0.5 h it took to reach the desired temperature in the steam chamber. Furthermore, the heat transfer may also be quicker when plants are immersed in water than when exposed to steam. In an experiment in Finland, where potted strawberry runners were exposed to a warming-up period of 30 min followed by 42 min at 43 °C and an RH close to saturation, *P. pallidus* was completely eradicated (Tuovinen et al. 2003), and a similar result was reported from California (USA) after 45 min steaming at 43.3 °C (Smith and Goldsmith 1936). Further experiments are needed, with a higher volume of mite-infested plant material, but there may be a potential to reduce the period of steaming even further in the production of strawberry mite free transplants. In contrast, the one egg still viable after 2-h exposure (including the 0.5 h warming-up), indicates that this is around the minimum required exposure time with the current steaming technology. Eggs of the strawberry mite are very small, around 0.125 × 0.075 mm (Alford 2014), and some of them may thus be protected by small hollows in the leaf surface or petioles for a certain time. However, in the present study, the killing effect on *P. pallidus* by steaming was similar for the leaves on crowns and on the single leaves picked in the field, and this indicates that even the innermost surfaces of folded leaflets were heated sufficiently.

Investigations in Switzerland reported the adult females to be the most heat tolerant stage of *P. pallidus* (Wiesmann 1941). A study in Norway found that, when immersed in 44 °C water, longer exposure time was needed to kill eggs (480 s) than adults (360 s); however, at higher temperatures adult females tolerated the heat treatment better than eggs (Stenseth 1975). Two stages were less thoroughly tested in the present study: adult males and pupae. A few males (about one per leaflet) were present in the first run only, and their mortality after 2- or 4-h steam treatments was 100%. Eradication of males is of minor importance in this facultatively parthenogenic species. The pupal stage of *P. pallidus* lasts for about 3 days at 20 °C (Smith and Goldsmith 1936; Wiesmann 1941). Thus, if pupae had survived the steam treatments to a significant degree, there would have been some live adults originating from them in the counts of mobile stages made 2–3 days after treatment. The continued development of surviving mites in the days between steaming and assessment also explains why mite counts tended to be higher in the control treatment than in the steam treatments.

The strawberry plants used to study the effect on *P. pallidus* in our study were collected in the field in mid to late August, at a time when the strawberry mite was still active. Adult *P. pallidus* collected from Swedish strawberry fields became more tolerant to hot water treatment at the end of the growing season, from mid-September throughout October, at a time when the mites prepare for hibernation (Hellqvist 2002). It could thus be expected that a longer exposure time is needed to eliminate the strawberry mite from cold stored runners than on fresh runners collected in the summer. The killing effect on *P. pallidus* of the steam treatment should therefore be further evaluated on strawberry plant material collected in late autumn, on cold stored strawberry transplants or on bare-root plants lifted from the ground in early spring, as suggested by Hellqvist (2002).

The present study clearly showed that even up to 6 h exposure to 44 °C in the steam chamber did not eradicate or severely suppress *T. urticae*. The survival of the steamed non-diapausing females was high, and they continued to produce eggs the following days. The hatchability of the eggs laid post-treatment was not studied but was possibly high as a high proportion of the steamed eggs hatched, and providing that the steaming did not cause heat-induced sterility in the females, as has been found for some insects (Hansen et al. 2011). The survival of larvae and nymphs that developed from steamed eggs was also very high, and there was no significant suppressive effect of steaming on the total population density of immature stages 5 or 6 days following the steaming. In a study in Florida in a similar steam chamber as used in the present work, 4 h treatment at 44 °C of Lima bean leaves infested with *T. urticae* only killed 15% of the non-diapausing adult females and 20% of the eggs (Renkema et al. 2020). Furthermore, the same study showed that strawberry transplants exposed to this treatment in comparison with untreated transplants did not have a lower spider mite density 7 weeks after planting.

As for *P. pallidus*, the time needed to eradicate *T. urticae* at a certain temperature is shorter when immersing infested planting material in water than exposing them to steam or dry air. In New Zealand, postharvest dipping of persimmon fruits infested with *T. urticae* in 44 °C water nearly eliminated non-diapausing and diapausing female adults after 102 and 211 min, respectively (Lester et al. 1997). Immersion of strawberry leaf discs infested with *T. urticae* in 47.5 °C water eliminated non-diapausing adult females in 20 min and eggs in 10 min in a trial in Japan (Gotoh et al. 2013), whereas it took 2 h to eradicate non-diapausing adult females and eggs of *T. urticae* on bean leaf discs in 48 °C aerated steam in Florida (Renkema et al. 2020). In a trial in New Zealand, it took 8.5 and 15 h to reach 50 and 99% mortality, respectively, of *T. urticae* females on nectarine fruits when kept in 45–46 °C air at approximately 60% RH (Waddel and Birtles 1992). Possible factors that might contribute to the accelerated mortality in hot-water treatments may be a more effective heat transfer to the mite body, as indicated by Renkema et al. (2020) and hypoxia, which likely limit the capacity of insects to tolerate heat stress (Harrison et al. 2018). Another factor affecting population sizes may also be a washing-off effect, and Gotoh et al. (2013) found that up to 39% of the females but no eggs were washed off strawberry leaf discs when immersing leaf material in water.

It has been shown that exposure of *T. cinnabarinus* (syn. *T. urticae*) to 34–40 °C for 1 h followed by recovery at 26 °C increased the expression levels of protective heat shock proteins (Feng et al. 2010; Li et al. 2009). It is thus not unlikely that the pre-treatment at 37 °C and the recovery period at ambient temperature in our study and by Renkema et al. (2020) have contributed to a higher heat tolerance in *T. urticae* than if treated with hot water without any pre-treatment (Lester et al. 1997; Gotoh et al. 2013).

Strawberry transplants may carry all life-stages of *T. urticae*, including diapausing females. Our study included only non-diapausing *T. urticae* females and eggs of one strain. Earlier studies on the effect of forced hot air and hot water treatments have shown that the lethal exposure time at temperatures between 43 and 48 °C was longer for diapausing females than for non-diapausing females (Waddell and Birtles 1992; Lester et al. 1997). The thermal response in *T. urticae* is complex and can be influenced by other environmental stress experienced prior to the heat treatment. Feng et al. (2010) showed that both a selected heat tolerant *T. urticae* strain and a strain resistant to the acaricide abamectin had higher expression levels of a heat shock protein than an acaricide susceptible strain with normal thermal sensitivity. This might imply an interaction between acaricide resistance status and heat tolerance, and that resistant mites may be more tolerant to heat. Moreover, tolerance to high temperature may differ between populations of *T. urticae* (Gotoh et al. 2013). Further evaluation of the effect of steam treatments should thus include different strains of *T. urticae* and diapausing females. The effect of steam treatments on larvae and nymphs of *T. urticae* have not been studied, but we might assume that they are less heat tolerant than the adults due to their smaller body size.

With the presently used steaming technology, a higher temperature than 44 °C or a longer exposure time than 6 h seems necessary to suppress adults and eggs of *T. urticae*. In the study with steam treatments in Florida, 4 h at 46 °C increased mortality in non-diapausing adults and eggs to 70 and 60%, respectively, and eradication or near eradication of these developmental stages was achieved in about 2 h at 48 °C (Renkema et al., 2020). However, there are clear indications that such exposure times at 46 and 48 °C may be devastating for the plant material (Turechek and Peres 2009; Turechek et al. 2021).

In this study, we only treated well-spaced single leaves or crowns stripped for much leaf material and all roots, which ensured good exposure of *P. pallidus* to the aerated steam. However, the technology is presently offered at a large-scale commercial size level that includes a vacuum technique that distributes steam rapidly and evenly at the desired temperature in dense loads of transplants (S. Myhrene, Moleda AS, pers. comm.). Based on previous studies of effects on growth and yield (Nielsen 2021; Turechek et al. 2021; Wang et al. 2019) and the present investigation, the steaming technology provides a great potential in eradication of the strawberry mite in the strawberry transplant production.

## Data Availability

The datasets generated and/or analyzed during the current study are available from the corresponding author on reasonable request.
